# Anabolic treatments for osteoporosis in postmenopausal women

**DOI:** 10.12703/r/10-44

**Published:** 2021-05-05

**Authors:** Neelam Hassan, Celia L Gregson, Jon H Tobias

**Affiliations:** 1 Translational Health Sciences, Bristol Medical School, University of Bristol, Musculoskeletal Research Unit, Southmead Hospital, Bristol, UK

**Keywords:** Anabolic, Osteoporosis, Teriparatide, Abaloparatide, Romosozumab, Postmenopausal

## Abstract

Antiresorptive agents are generally recommended as first-line treatment for osteoporosis in postmenopausal women. These drugs suppress bone resorption but do not rebuild bone, limiting their efficacy. Antiresorptive use is further hampered by concerns over rare side effects, including atypical femoral fractures and osteonecrosis of the jaw. Anabolic treatments overcome limitations of antiresorptive treatment by stimulating new bone formation, reducing the risk of fracture with greater efficacy. This review summarises the latest trial data for the three anabolic agents currently available for the treatment of osteoporosis in postmenopausal women: teriparatide, abaloparatide, and romosozumab. Data from head-to-head studies comparing anabolic and antiresorptive treatments are reviewed. At present, anabolic treatments are generally reserved for use in patients with severe osteoporosis at very high fracture risk; the factors limiting their more widespread use are discussed together with how this may change in the future.

## Introduction

In osteoporosis, loss of bone tissue occurs, leading to skeletal fragility and an increase in fracture risk^1^. One in three women and one in five men over the age of 50 will sustain an osteoporotic fracture at some point in their lifetime^2^. Fractures of the hip and spine in particular are associated with high levels of morbidity and mortality^3–5^. Antiresorptive treatments, such as oral (e.g. alendronic acid and risedronate) and intravenous (e.g. zoledronate) bisphosphonates and subcutaneous denosumab, are the most widely used antiresorptive treatments for osteoporosis, reducing the risk of vertebral and hip fractures by 40–70% and 40–53%, respectively^6–11^. Their use is limited for several reasons^12^. Oral bisphosphonates are commonly associated with side effects such as gastrointestinal disturbance, which, together with difficult dosing regimens, contributes to the poor adherence rates seen^13^. In addition, suppression of bone resorption is associated with rare but serious side effects such as osteonecrosis of the jaw (ONJ) and, when prolonged, atypical femoral fractures (AFFs). Finally, since previously lost bone cannot be restored by antiresorptive agents, only finite gains in bone mineral density (BMD) can be achieved, limiting efficacy in severely affected individuals.

Anabolic treatments for osteoporosis, which act to stimulate bone formation, may overcome these limitations. At least theoretically, these agents are not associated with an increased risk of ONJ or AFF, are not limited in terms of BMD increment, and restore previously lost bone, making them effective in patients with severe osteoporosis. Here, we summarise the trial data for the three anabolic agents currently available for the treatment of osteoporosis in postmenopausal women, namely teriparatide, abaloparatide, and romosozumab, and consider their use in clinical practice.

## Teriparatide

### Trial data

Teriparatide (PTH 1-34) is a recombinant fragment of parathyroid hormone (PTH). It was developed after rats exposed to intermittent PTH were found to show a predominant bone formation response^14^. This contrasted with increased bone resorption and bone loss following prolonged continuous exposure to PTH, as occurs in patients with primary hyperparathyroidism^15^. This observation eventually led to the Fracture Prevention Pivotal Trial, published in 2001, in which postmenopausal women with a vertebral fracture were assigned to daily subcutaneous injections of 20 µg or 40 µg of teriparatide or placebo for 21 months^16^. Following the 20 µg dose of teriparatide, risks of vertebral and nonvertebral fracture were found to be reduced by 65% and 53%, respectively, compared with placebo.

Subsequently, in a head-to-head trial, teriparatide was found to be superior to alendronate at increasing BMD at the spine and hip, although the more clinically relevant outcome of fracture risk was not investigated^17^. However, teriparatide was found to reduce fracture risk in glucocorticoid-induced osteoporosis by 90% compared to alendronate over 18 months^18^. Another trial demonstrated that teriparatide reduced vertebral fractures by 50% compared to risedronate over 12 months in patients with acute painful vertebral fractures^19^. Although, of note, fracture outcomes were not the primary endpoints in any of these studies. More recently, the VERO trial investigated the effect of teriparatide vs. risedronate on incident radiographic vertebral fracture in women with severe osteoporosis^20^. After 24 months, there was a relative risk reduction of 56% in the teriparatide arm compared to the risedronate arm. The incidence of nonvertebral and clinical fractures was also reduced in the teriparatide arm by 34% and 52%, respectively. Recently, a systematic review and meta-analysis of 23 teriparatide trials determined specific efficacy against hip (but not upper limb) fractures of 56% in patients with osteoporosis^21^.

### Limitations and clinical use

Teriparatide is thought to be generally safe, though treatment duration is limited to 24 months because of a theoretical risk of osteosarcoma that was seen in rodents receiving high-dose teriparatide^22^. That said, this side effect has not been observed in humans in post-marketing surveillance^23^. In terms of side effects, 3% of patients suffer from persistent hypercalcaemia, and transient bone loss may occur at cortical bone sites such as the distal forearm^16^, both of which result from teriparatide’s co-stimulatory action on bone resorption.

In common with other anabolic agents, teriparatide is given by subcutaneous injection and must be stored in a refrigerator. Since the injection is administered on a daily basis, this can reduce patient uptake and adherence^24^. Attempts to develop alternative routes of administration have thus far been unsuccessful. The uptake of teriparatide has also been limited in many countries owing to its relatively high cost. This is likely to improve following the recent development of generic teriparatide and biosimilars. Four teriparatide preparations are now available for use in the UK, including two teriparatide biosimilars and a chemically synthesized generic version of teriparatide, which was launched in 2020. A further strategy to limit costs is to reduce the frequency of administration to once or twice weekly, which is also associated with gains in BMD, though to a lesser extent than that observed with daily treatment^25,26^.

Teriparatide is mainly used as a second-line treatment in severe osteoporosis after the failure of bisphosphonates or denosumab^27^. Concern has been raised as to whether this sequence might be detrimental, given teriparatide stimulates bone formation, which is reduced by antiresorptive agents as a result of coupling of bone formation to resorption. Transient hip BMD loss is reported for at least a year in patients transitioning from antiresorptives to teriparatide, particularly post denosumab^28^, suggesting teriparatide should ideally be used as a first-line treatment for severe osteoporosis to optimise its anabolic effect.

In patients who are already on an antiresorptive, continuing the antiresorptive whilst adding in teriparatide may be an effective strategy to mitigate bone loss. The DATA-Switch study, published in 2015, examined the effect on BMD of (a) 24 months of teriparatide followed by 24 months of denosumab, (b) 24 months of both teriparatide and denosumab followed by 24 months of denosumab only, and finally (c) 24 months of denosumab followed by 24 months of teriparatide^29^. Switching from teriparatide to denosumab resulted in BMD continuing to increase, whereas switching from denosumab to teriparatide resulted in progressive or transient bone loss. The highest increases in femoral neck and total hip BMD were seen in those receiving combination therapy, although this was not significantly different from the teriparatide to denosumab arm. A recent systematic review and meta-analysis of 19 trials also showed that combination therapy (i.e. teriparatide plus most commonly a bisphosphonate) was superior to teriparatide alone in terms of increasing BMD^30^, which may be explained by the bisphosphonate ameliorating teriparatide-induced increases in cortical porosity^28^.

Importantly, whether BMD losses seen following switch from antiresorptives to teriparatide translates to a reduction in anti-fracture efficacy remains unclear, as most studies examined BMD as the primary outcome rather than fracture risk. Of note, the VERO trial demonstrated substantial anti-fracture efficacy of teriparatide, even though 59% of patients in the teriparatide arm had previously been treated with bisphosphonates. In subgroup analyses, no evidence was seen for a difference in the anti-fracture efficacy of teriparatide in treatment-naïve patients vs.** those who had previously been treated with bisphosphonates^31^.

A further consideration is that cessation of teriparatide treatment may be followed by relatively rapid BMD loss^32^. It is now standard practice to follow a 2-year course of teriparatide with an antiresorptive agent to maintain BMD gains^29,32^, though the antiresorptive best suited for this purpose remains unclear. An alternative strategy would be to repeat the teriparatide treatment course. However, concerns about osteosarcoma have limited such investigation, although it is almost certain that these concerns have been overstated when compared to real-world human data^23^. To date, only one study has attempted to investigate teriparatide re-treatment after an initial 2-year course^33^; 30 µg of teriparatide was administered to both men and women with osteoporosis for 24 months, then withdrawn for a “drug holiday” of 12 months, before being reintroduced for another 12 months. The response to teriparatide re-treatment, in terms of BMD gain and increases in bone turnover markers, was significantly attenuated compared to the initial course. Although retreatment restored BMD to pre-drug holiday levels, there was no further increase in BMD. This resistance to retreatment with teriparatide remains unexplained, although various mechanisms have been postulated, such as receptor downregulation or increase in negative regulators of bone formation, e.g. Dickkopf-1 (Dkk1)^34^.

## Abaloparatide

### Trial data

Abaloparatide is a synthetic peptide analogue of human PTH-related protein, developed with the aim of decoupling PTH’s anabolic action from stimulation of bone resorption, thereby reducing the risk of adverse effects such as hypercalcaemia, and avoiding transient bone loss at cortical sites as seen with teriparatide. Abaloparatide and teriparatide have differences in affinity for the two stable conformations (RG and R^0^) of the PTH1 receptor^35^. Abaloparatide has a 1,600-fold greater affinity for the R^0^ conformation of the receptor compared with teriparatide. It is postulated that abaloparatide’s greater affinity for the R^0 ^conformation results in a greater increase in bone formation than bone resorption.

Abaloparatide was approved for use in the USA for the treatment of postmenopausal women with osteoporosis at high risk of fracture in 2017, following the Abaloparatide Comparator Trial in Vertebral Endpoints (ACTIVE) trial^36^. This was a phase III, double-blind, three-arm study in which postmenopausal women with osteoporosis were randomised to receive 80 µg daily subcutaneous abaloparatide, 20 µg daily subcutaneous teriparatide, or placebo for 18 months. Those receiving abaloparatide had an 86% relative risk reduction in vertebral fractures compared to placebo, with a 43% relative risk reduction in nonvertebral fractures. BMD at the lumbar spine, femoral neck, and total hip increased rapidly compared to placebo at 6, 12, and 18 months. Though teriparatide led to a slightly smaller reduction in nonvertebral fractures (28%) overall, abaloparatide and teriparatide showed similar anti-fracture efficacy. There was a lower incidence of 4-hour post-dose hypercalcaemia with abaloparatide compared to teriparatide; however, serious adverse events were similar across the three arms of the trial. That said, there were more adverse events leading to discontinuation after abaloparatide (9.9%) than teriparatide (6.8%) or placebo (6.1%). In the subsequent ACTIVExtend study, women who had completed the original ACTIVE study were treated with 24 months of alendronate^37^. There was a relative risk reduction of vertebral (39%), clinical (34%), and major osteoporotic fractures (50%) in the abaloparatide to alendronate arm vs. the placebo to alendronate arm.

### Limitations and clinical use

As a high-cost daily subcutaneous injection, many of the limitations of teriparatide are shared by abaloparatide. Although abaloparatide was approved for use in the USA in 2017, in 2018 the European Medicines Agency refused marketing approval in the European Union (EU). Poor compliance to good clinical practice at two study sites, the inability of the study documentation to convincingly demonstrate efficacy for nonvertebral fracture prevention, and an observed increase in palpitations and heart rate were cited as reasons for this rejection^38^.

## Romosozumab

### Trial data

Romosozumab, a monoclonal antibody against sclerostin, was developed as an anabolic osteoporosis treatment following the discovery that loss-of-function mutations in the *SOST* gene, which produces sclerostin, underlies the rare high bone mass disorder sclerosteosis^39^. In contrast to the PTH analogues, romosozumab stimulates bone formation but also simultaneously reduces bone resorption^40^. Romosozumab was recently approved as a new anabolic osteoporosis treatment in the USA and in Europe following two phase III trials confirming its anti-fracture efficacy.

The FRAME trial investigated the effect of monthly subcutaneous romosozumab 210 mg vs. placebo on fracture risk^41^. After 12 months, romosozumab conveyed 73%, 36%, and 25% relative risk reductions in vertebral fractures, clinical fractures, and nonvertebral fractures, respectively, although the relative risk reduction for nonvertebral fracture was weak (relative risk [RR] 0.75 [95% confidence interval (CI) 0.53–1.05], *P* = 0.1). However, in post-hoc analyses, excluding the low-risk Colombian cohort, there was stronger evidence of a decrease in nonvertebral fracture risk in the romosozumab arm with a relative risk reduction of 42% (*P* = 0.01)^42^. Romosozumab resulted in a marked gain in BMD (lumbar spine BMD increased by 13% over 12 months), with bone turnover markers indicating this was due to a combination of increased bone formation and reduced bone resorption. In the FRAME Extension study, women who had received subcutaneous romosozumab or placebo for 12 months were then switched to open-label subcutaneous denosumab every 6 months for 24 months^43^, after which a relative risk reduction in new vertebral fractures and nonvertebral fractures persisted in the romosozumab arm compared to the placebo arm. Moreover, the increase in BMD in the first 12 months of the romosozumab arm was maintained over the subsequent 24 months during treatment with denosumab, after which on average 10.5% and 5.2% increases in lumbar spine and total hip BMD, respectively, were seen.

The subsequent ARCH study investigated the effectiveness of a sequential treatment regimen consisting of anabolic romosozumab consolidated by the antiresorptive alendronic acid^44^. ARCH was a randomised double-blind multicentre trial that compared romosozumab for 12 months followed by transition to alendronic acid for 12 months vs. alendronic acid alone for 24 months. In contrast to the FRAME trial, the study population was of relatively high fracture risk (96% had had a vertebral fracture, 9% a recent hip fracture). Fracture risk reductions were apparent at 12 months in the romosozumab arm (37% reduction in vertebral fractures, 28% in clinical fractures, and 26% in nonvertebral fractures). By 24 months, there was a relative risk reduction of 48% in new vertebral fractures, 27% in clinical fractures, 19% in nonvertebral fractures, and 38% reduction in hip fractures in those receiving romosozumab then alendronic acid vs. alendronic acid throughout.

### Limitations and clinical use

Though romosozumab is associated with a marked anabolic response, this appears to be relatively short lived. For example, in the FRAME study, while the osteoblast marker P1NP initially increased in the romosozumab group by approximately 150%, it had normalised by 9 months^41^. To ensure sufficient trial duration to evaluate effects on fracture risk, a sequential therapy was needed, whereby romosozumab was followed by an antiresorptive agent. The same is also likely to apply to clinical use of this treatment.

The effect of a second course of romosozumab was investigated in an extension of the phase II dose-finding study^45^. Postmenopausal women with low BMD (T-score ≤−2.0 and ≥−3.5) received romosozumab or placebo from months 0–24, followed by placebo or denosumab from months 24–36, and then romosozumab from months 36–48. The second course of romosozumab resulted in further BMD increases of 12.4% at the lumbar spine and 6% at total hip in the romosozumab-placebo-romosozumab arm, similar to the BMD increases seen with the initial course (12% and 5.5%, respectively). In the romosozumab-denosumab-romosozumab arm, the second course of romosozumab generated smaller BMD gains (2.3% at the lumbar spine, unchanged at total hip), although the second romosozumab course appeared to counteract the BMD loss that would be expected after discontinuation of denosumab, contrasting with what is seen with the switch from denosumab to teriparatide^29^. Romosozumab’s action, of reducing bone resorption as well as stimulating bone formation, is likely to account for this difference in response. It is important to note that fracture risk reduction was not assessed, so it is unclear whether repeated romosozumab treatment courses would enhance anti-fracture efficacy.

Whilst no safety signals were seen in the FRAME study, an increase in major adverse cardiovascular events (MACE) was noted in the romosozumab arm over the first 12 months in the ARCH study (2.5% vs. 1.9% in the alendronic acid arm). A recent systematic review and meta-analysis investigating the effect of romosozumab on cardiovascular outcomes demonstrated a 39% increase in four-point MACE (including death, myocardial infarction, stroke, and heart failure) amongst postmenopausal women and older men with osteoporosis over a period of 12–36 months^46^. This finding was again largely driven by the results of the ARCH trial.

Several hypotheses have been suggested to explain this difference^47,48^. As ARCH focussed only on women with severe osteoporosis, the study population was older in comparison to that of FRAME, with a higher baseline prevalence and risk of cardiovascular disease. However, there was no imbalance of cardiovascular risk factors between the romosozumab and alendronic acid arms. There is some evidence that those genetically predisposed to lower sclerostin expression in bone have a greater risk of major adverse cardiovascular events^49^; however, to what extent this predicts the effect of sclerostin inhibitors on vascular tissue remains unclear. Another possible explanation is that the ARCH study used a bisphosphonate as the comparator; a previous trial found that 18-monthly zoledronate over 6 years reduced all-cause mortality, possibly indicating a protective effect of bisphosphonates on risk of cardiovascular events rather than an adverse effect of romosozumab^50,51^. However, this has not been borne out in a subsequent systematic review and meta-analysis of 38 randomised clinical trials^52^. The alternative possibility is that the difference in rates of MACE observed in the ARCH trial was due to chance, particularly as no effect was observed in the placebo-controlled FRAME trial^48^.

Nonetheless, concern about the cardiovascular safety profile led to romosozumab being given only restricted approval to selected groups with severe osteoporosis by the European Medicines Agency in October 2019 after initially being refused.

## When should an anabolic agent be prescribed for osteoporosis?

As discussed above, anabolic drugs for osteoporosis serve to increase BMD and reduce fracture risk quickly and to a greater extent than both placebo and conventional antiresorptive therapies (Table 1). Increases in BMD, particularly at the hip, appear to be greatest when anabolic agents are used *de novo* rather than after antiresorptive use. It is therefore attractive to consider using anabolic agents as first-line treatment in certain patient groups. For example, since the anti-fracture efficacy of anabolic agents appears to be greatest at the spine, it may be that these agents are best used in those at high risk of vertebral fractures. Another strategy is to target those at very high risk of imminent fracture, defined as a high risk of fracture within the next 12–24 months. It can be difficult to determine precisely which individuals are at imminent risk of fracture. Prior fracture type, age, sex, falls risk, comorbidities, and medications can all increase individual fracture risk^53^. However, recency of prior fracture has been found to be the key predictor of imminent fracture risk^54^. The risk of a second major osteoporotic fracture is highest immediately after the first fracture, and most subsequent osteoporotic fractures occur within 5 years of the initial fracture^55–57^.

**Table 1.** Effect of anabolic treatments on vertebral and nonvertebral fracture risk in postmenopausal women with osteoporosis.

Drug	Trial	Comparator	Duration (months)	Vertebral fracture RRR	Non-vertebral fracture RRR
**Teriparatide**	FPT^16^	Placebo	21	65%	53%
	VERO^20^	Risedronate	24	56%	34%
	ACTIVE^36^	Placebo	18	80%	28%^b^
**Abaloparatide**	ACTIVE^36^	Placebo	18	86%	43%
**Romosozumab**	FRAME^41^	Placebo	12	73%	25%
	ARCH^44^^a^	Alendronic acid	24	37% (12 months) 48% (24 months)	26% (12 months) 19% (24 months)

^a^Romosozumab for 12 months followed by alendronic acid for 12 months vs. alendronic acid for 24 months. ^b^Non-significant (relative risk 0.72 [95% confidence interval 0.42–1.22], *P* = 0.22). RRR = relative risk reduction

Cut-offs for identifying individuals with a very high fracture risk as assessed using the FRAX algorithm have recently been proposed, which take into account recency of fracture^58,59^. An important consideration is that current fracture risk calculators such as FRAX are primarily geared towards predicting hip fractures rather than vertebral fractures. This has the advantage that greater weighting is applied to hip fractures, the most costly in terms of healthcare spending, than other fractures. However, although anabolic drugs reduce hip fracture risk, they are more efficacious at reducing the risk of vertebral fractures, reflecting the fact that hip fractures arise from multiple factors beyond low BMD^2^.

Despite demonstrating greater clinical efficacy than antiresorptives, currently teriparatide is the only anabolic drug for osteoporosis available through the National Health Service (NHS) in the UK, where its use is restricted by high cost; the same is also expected to apply to romosozumab. In the UK, access to high-cost medicines is governed by the National Institute for Health and Care Excellence (NICE), with recommendations largely based on considerations of cost effectiveness. NICE last reviewed the use of teriparatide in 2018^60^; more recently, both teriparatide and romosozumab underwent a full economic evaluation as part of a National Institute for Health Research (NIHR)-funded Health Technology Assessment (HTA)^61^, which is likely to be considered when NICE next review these treatment options. This HTA found that although both teriparatide and romosozumab are highly effective at preventing fragility fractures, the incremental cost-effectiveness ratios (ICERs) for both interventions exceed the commonly applied threshold of £30,000 per quality-adjusted life-year (QALY) compared with no treatment across a range of probabilities for fracture risk. The authors noted that estimates given of incremental net monetary benefit in patients at very high fracture risk were uncertain, as they were based on only a very small proportion of the simulated population. The cost-effectiveness specifically in individuals at high risk of vertebral fractures was not examined; therefore, it remains unknown whether these anabolic agents may indeed be cost-effective in this population. Whilst hip fractures continue to be the main driver in cost-effectiveness analyses, the use of anabolic drugs in cost-limited systems such as the NHS is likely to continue to be restricted.

A systematic review of cost-effectiveness analyses for osteoporosis drugs across 15 countries was recently updated to include emerging data on sequential therapy starting with teriparatide or abaloparatide followed by alendronic acid compared with no treatment, placebo, or alendronic acid alone^62^. The results from the three studies included in the analysis were mixed, with ICERs strongly affected by the very high costs of abaloparatide and teriparatide. One study indicated that the cost of generic teriparatide or biosimilars would need to be 65–85% lower than the originator for sequential treatment to be cost-effective^63^. A more recent study assessing the cost-effectiveness of sequential treatment starting with romosozumab followed by alendronic acid compared to alendronic acid alone for postmenopausal women at high risk of fracture in Sweden demonstrated an ICER of €33,732, well below the Swedish threshold of €60,000 per QALY^64^.

The development and marketing of generic teriparatide and biosimilars may offer some hope of re-appraising the cost-effectiveness equations. To enable cost-effectiveness models that are more suitable for evaluating anabolic drugs for osteoporosis, and to define groups to whom these should be offered, further work is needed to improve our understanding of the economic consequences of vertebral fractures and to develop more accurate strategies for individualised vertebral fracture risk assessment.

## Conclusions

Therapeutic options for osteoporosis have recently increased with the availability of new anabolic therapies: teriparatide, which has been available for many years, increases both bone formation and bone resorption; abaloparatide (not licensed in Europe) acts in a similar manner to teriparatide but may have less tendency to stimulate bone resorption; romosozumab increases bone formation whilst reducing bone resorption. However, though overcoming limitations of antiresorptive treatment in terms of clinical efficacy and rare side effect risks, anabolic treatment use is likely to remain limited by cost in particular. The parenteral route of administration required for all currently available anabolic agents may also limit patient uptake and adherence. Looking to the future, it may be possible to expand the use of these drugs through greater understanding of which patients are likely to benefit most and, in the case of teriparatide, by the emergence of biosimilars. Greater understanding is also needed as to how best to integrate these treatments with conventional antiresorptive agents in the form of sequential or even combination therapy. The benefit of repeated treatment courses also warrants further examination, with a focus on the clinically relevant outcome of fracture risk reduction rather than solely changes in BMD.
